# Application of Viscose-Based Porous Carbon Fibers in Food Processing—Malathion and Chlorpyrifos Removal

**DOI:** 10.3390/foods12122362

**Published:** 2023-06-13

**Authors:** Tamara Tasić, Vedran Milanković, Katarina Batalović, Stefan Breitenbach, Christoph Unterweger, Christian Fürst, Igor A. Pašti, Tamara Lazarević-Pašti

**Affiliations:** 1 VINČA Institute of Nuclear Sciences—National Institute of the Republic of Serbia, University of Belgrade, Mike Petrovica Alasa 12–14, 11000 Belgrade, Serbia; tamara.tasic@vin.bg.ac.rs (T.T.); vedran.milankovic@vin.bg.ac.rs (V.M.); kciric@vin.bg.ac.rs (K.B.); tamara@vin.bg.ac.rs (T.L.-P.); 2 Wood K Plus—Kompetenzzentrum Holz GmbH, Altenberger Strasse 69, 4040 Linz, Austria; s.breitenbach@wood-kplus.at (S.B.); c.unterweger@wood-kplus.at (C.U.); c.fuerst@wood-kplus.at (C.F.); 3 Institute of Chemical Technology of Inorganic Materials (TIM), Johannes Kepler University Linz, Altenberger Strasse 69, 4040 Linz, Austria; 4 Faculty of Physical Chemistry, University of Belgrade, Studentski Trg 12–16, 11158 Belgrade, Serbia

**Keywords:** biomass, biowaste, activated carbon materials, pesticides, organophosphates, adsorptive removal, properties–performance relations

## Abstract

The increasing usage of pesticides to boost food production inevitably leads to their presence in food samples, requiring the development of efficient methods for their removal. Here, we show that carefully tuned viscose-derived activated carbon fibers can be used for malathion and chlorpyrifos removal from liquid samples, even in complex matrices such as lemon juice and mint ethanol extract. Adsorbents were produced using the Design of Experiments protocol for varying activation conditions (carbonization at 850 °C; activation temperature between 670 and 870 °C; activation time from 30 to 180 min; and CO_2_ flow rate from 10 to 80 L h^−1^) and characterized in terms of physical and chemical properties (SEM, EDX, BET, FTIR). Pesticide adsorption kinetics and thermodynamics were then addressed. It was shown that some of the developed adsorbents are also capable of the selective removal of chlorpyrifos in the presence of malathion. The selected materials were not affected by complex matrices of real samples. Moreover, the adsorbent can be regenerated at least five times without pronounced performance losses. We suggest that the adsorptive removal of food contaminants can effectively improve food safety and quality, unlike other methods currently in use, which negatively affect the nutritional value of food products. Finally, data-based models trained on well-characterized materials libraries can direct the synthesis of novel adsorbents for the desired application in food processing.

## 1. Introduction

With an increasing global population, the demand for food is also rising [1]. Farmers and producers must find ways to enhance food production to meet current needs. This can be accomplished through various techniques, such as improving crop yields, diversifying crop varieties, reducing food waste and loss, and improving agricultural methods [2,3].

The Farm to Fork Strategy is an economic, environmental, and social policy initiative to transform and modernize the EU’s food system [4]. It will enable the EU to become a more sustainable, resilient, and competitive food system while improving the quality and safety of the food we consume and reducing our environmental footprint. Some of Farm to Fork’s key focal points are promoting sustainable consumption, stimulating the production of more nutritious and safe food, investing in research and innovation, and stimulating the shift toward ecological farming. One of the core objectives of the Farm to Fork Strategy is to reduce the use and risk of chemical pesticides and other synthetic substances in food production. To achieve this, the EU promotes more sustainable alternatives to chemical pesticides, such as integrated pest management, biocontrols, and organic farming. The Farm to Fork strategy also aims to ensure that any chemical pesticides used on food crops are in line with EU standards, which include an increased focus on the safety and protection of the environment.

Pesticides are chemical substances that kill or control insects, weeds, fungi, bacteria, and other organisms that can damage crops or transmit diseases to humans [5]. They vary in their mode of action, toxicity, persistence, and environmental impact. Using pesticides in food plant production is necessary for controlling pests, weeds, and diseases that can adversely affect plant yields. Organophosphate pesticides (OPs), such as malathion and chlorpyrifos, are commonly used to control pests on fruit crops. They are used on various fruits, such as apples, oranges, lemons, limes, peaches, nectarines, bananas, grapes, and watermelons. The use of OPs is highly regulated due to their ability to inhibit the activity of acetylcholinesterase (AChE) [6,7,8,9]. AChE is an important enzyme in the nervous system, and disruption of its activity leads to many health issues, even death [10]. Malathion and chlorpyrifos are used worldwide and, hence, are often found in food samples. While chlorpyrifos is well known to be highly poisonous [11], malathion is misleadingly considered moderately toxic. These estimations refer to the acute toxicity of the mentioned pesticides, while recent research indicates that these compounds are potentially neurotoxic, even in small amounts [6,7,12].

Food processing is any process or method used to transform raw ingredients into safe, edible, and shelf-stable food products [13]. Fruit and spice extracts are popular ingredients in the food industry [14]. They are made by extracting juice, essential oils, pigments, and other components from fresh fruits, such as lemon or mint. These extracts are used to enhance the taste, color, and texture of food products and provide antioxidants and other health benefits [15]. As such, they are often found in food products such as juices, yogurts, ice creams, gums, and baked goods. Because the plants used for extract production are often exposed to pesticides, the final products are also expected to contain their toxic residues.

The use of pesticides has been debated for decades, as many people are concerned about the potential long-term effects of these chemicals on our health and the environment [16]. Pesticide use is necessary for food production to protect crops from pests and diseases. However, the extensive use of pesticides can lead to pesticide accumulation, which can be detrimental to human health due to toxicity [17]. Because we still cannot avoid using them, the solution to the problem may be to act on the level of food processing [18].

Pesticides can be removed from samples using chemical, physical, or biological methods [19]. As an additional step in food processing, adsorption has the best potential. In terms of pesticide remediation, adsorption is a process of removing pesticide molecules from various samples by attaching them to an adsorbent material [20]. The adsorbent materials most commonly used for pesticide removal include activated carbon materials [20,21], mesoporous monetite [22], porous metal–organic frameworks [23], mineral surfaces [24], organohydrotalcite [25], zeolites [26], materials from the graphene family [27], metal nanoparticles [28], and others. Activated carbon materials have an excellent potential for removing pesticides during food processing. They are highly porous, cheap, available, and easy to use. In addition, they are mostly non-toxic, so their use is safe [29]. Moreover, carbon materials produced from biomass represent a sustainable choice for pesticide removal. The activated carbon materials can be produced by the pyrolysis of different biomass materials, such as wood, coconut husks, seed hulls, and other organic waste materials [30,31,32]. The activated carbon material has a high surface area, which allows it to absorb and remove many organic contaminants effectively.

Viscose fibers are a type of fiber made from cellulose that are commonly studied as a precursor for producing activated carbon materials. These fibers can be used in a variety of applications, including the removal of several pollutants. Plens et al. used activated carbon materials derived from viscose fibers for nitrogen (NOx) and sulfur (SOx) adsorption [33]. Bhati et al. were able to use carbonized viscose fiber for the effective removal of iodine and CCl_4_ [34]. It was also successfully used for the removal of heavy metals [35], dyes [36], and wastewater treatment [37]. In our previous paper, we used viscose fibers impregnated with diammonium hydrogen phosphate to efficiently remove different OPs from an aqueous solution [20,21,38]. A well-defined series of materials with gradually changing properties, such as pore size distribution, pore volume, and chemical composition, allowed for the analysis of the interconnection between the physico-chemical properties of adsorbents and their performance for OP removal.

Methods currently used during food processing with the aim of removing present contaminants include various chemical and physical techniques. Bleaching, UV radiation, and chemical treatment are commonly applied but severely affect the nutritional values of the products. We aimed to show an alternative filtration method for pesticide removal without the essential nutrient reduction.

This paper investigates the potential of using viscose-based porous carbon fibers as adsorbents for malathion (aliphatic OP) and chlorpyrifos (aromatic OP) removal considering fundamental and practical aspects. The main focus is on the investigated materials’ application in food processing—the removal of malathion and chlorpyrifos residues from lemon and mint extracts. First, the series of carbon materials used as adsorbents were synthesized using the Design of Experiments (DoE) protocol [39], characterized in terms of morphology, chemical composition, and textural properties, and then the kinetics and thermodynamics of malathion and chlorpyrifos adsorption from aqueous solutions were addressed. Next, the materials performance was linked to the synthesis conditions and their properties using principal component analysis and principal component regression. Then, the practical applicability of the presented series of materials was addressed, considering food safety and sustainability. Finally, the feasibility and potential benefits of using the adsorptive removal of OPs in food processing are discussed.

## 2. Materials and Methods

### 2.1. Material Synthesis

Viscose fibers (1.7 dtex, 38 mm) provided by Lenzing AG (Lenzing, Austria) were used as the precursor. They were washed thoroughly with distilled water before use. The viscose fibers were centrifuged with a spin dryer for 15 min before being dried for 24 h at 90 °C in a drying cabinet. The residual moisture was determined with a moisture analyzer (MX-50, A&D Company, Tokyo, Japan) at 105 °C until the mass remained constant. The fibers were only used if the residual moisture was below 4.5%.

The carbonization of the precursor was carried out by loading 100–400 g into a chamber furnace (HTK 8 W, Carbolite Gero GmbH, Neuhausen, Germany). After the evacuation of the chamber, a nitrogen flow atmosphere of 250 L h^−1^ was established. The sample was heated to 850 °C at a 1.0 °C/min heating rate and held isothermal for 30 min. It was then cooled to room temperature under a nitrogen atmosphere.

Parametric space for carbonized viscose fibers activation was generated using the DoE approach. The three independent variables, the activation temperature, activation time, and CO_2_ flow rate, were systematically varied each at three different levels using a Central Composite Design (CCD). As no significant differences in the carbon yield and porosity parameters could be measured, no replicates of the center point Run10 are included in this study (see Table 1 for details). The levels of the three variables were chosen based on preliminary tests. A temperature of 870 °C was found to be the highest suitable activation temperature, as further increase resulted in the complete consumption of the samples due to the fast kinetics of the activation reaction. An activation time of 105 min and a CO_2_ flow rate of 45 L h^−1^ were found suitable in preliminary tests and used as center points in our CCD. The lower and upper limits were chosen in order to ensure the start of the activation process at the lower limits and avoid the complete consumption of the sample at the upper limits. Activation was performed in a rotary kiln (RSR-B 120/500/11, Nabertherm GmbH, Lilienthal, Germany). For the activation, 10 g of the sample was placed in the middle of the quartz glass reactor. Prior to use, N_2_ was used to purge any air in the setup at a flow rate of 100 L h^−1^. Subsequently, the materials were heated from room temperature to the desired final activation temperature under an N_2_ flow rate of 50 L h^−1^. The sample was kept isothermally under the N_2_ flow for 30 min to ensure that the temperature was uniform throughout the reaction chamber. Afterward, the N_2_ flow was terminated and replaced by CO_2_ at the desired flow rate for a specified amount of time (Table 1). The activation process was completed by the termination of the CO_2_ flow and restarting of the N_2_ flow (50 L h^−1^) until the kiln was cooled to room temperature.

### 2.2. Material Characterization

A scanning electron microscope (SEM) PhenomProX (Thermo Fisher Scientific, Waltham, MA, USA) was used to investigate the samples’ morphology and elemental composition using Energy-Dispersive X-ray Analysis (EDX).

N_2_ isothermal adsorption (−196.15 °C) on a gas sorption system (Autosorb-iQ, Anton Paar QuantaTec Inc., Graz, Austria) was employed to analyze the specific surface area and textural structure of the activated carbon samples. Before the analysis, the samples were de-gassed for at least 2 h at 200 °C. The specific surface area was calculated using the method of Brunauer–Emmett–Teller (BET) [40], while the non-local density functional theory (NLDFT) was applied for derived pore size distribution (PSD) calculations.

A Nicolet iS20 FT-IR spectrophotometer (Thermo Fisher Scientific, Waltham, MA, USA) was used for the FTIR spectra recording. The applied wavenumber range was from 4000 to 500 cm^−1^ with 64 scans and 4 cm^−1^ resolution.

### 2.3. Adsorption Experiments

Adsorption experiments were carried out in batch (stationary conditions) and filter (dynamic conditions). For stationary analysis, prepared activated carbon fibers were first dispersed in double-distilled water. To provide the targeted concentration of adsorbent and OPs (malathion and chlorpyrifos), the desired amount of 10^−1^ mol dm^−3^ OP stock solution (Pestanal, Sigma Aldrich, Søborg, Denmark) was added. A laboratory shaker (Orbital Shaker-Incubator ES-20, Grant-Bio, Cambridgeshire, UK) was used for the adsorbent + OP mixture shaking and incubation at 25 °C for desired times (from 1 to 60 min). The adsorbent + OP mixture was centrifuged for 10 min at 14,500× *g* after the incubation. Next, the nylon filter (pore size 220 nm KX Syringe Filter, Kinesis, Cole Parmer, St. Neots, UK) was used for the supernatant filtration. The filtrate was subjected to ultra-performance liquid chromatography (UPLC) analysis, as described below, in order to determine the OP concentration after the adsorption. Modified commercial filters were used to analyze the OP adsorption onto the investigated materials under dynamic conditions. A total amount of 1 mg of each material was dispersed in 1 mL of deionized water and injected into the commercial nylon filter (pore size 220 nm KX Syringe Filter, Kinesis, Cole Parmer, St. Neots, UK). Compressed air was used for excess water removal from the modified filter. Next, 1 mL of the desired final concentration OP solution was injected through the modified filter with a flow rate of 1 mL min^−1^. The filtrate was subjected to UPLC analysis, as described below. Modified filters were disassembled after the experiments to check for the uniformity of the carbon material layer. Uniform distribution of the adsorbent over the nylon membrane was observed in all cases.

The adsorption efficiency was calculated as Uptake = 100% × (*C*_0_ − *C*_eq_)/*C*_0_ (*C*_0_—the starting concentration of OPs) for stationary or dynamic experiments. UPLC was used to determine the concentration of OPs in filtrates after adsorption (*C*_eq_). To confirm that there was no OP degradation during batch experiments, control experiments were performed in identical ways but without carbon materials.

A Waters ACQUITY UPLC system with a photodiode array (PDA) detector, controlled by Empower software, was used for OP analysis. An ACQUITY UPLC™ BEH C18 column (1.7 μm, 100 mm × 2.1 mm, Waters GmbH, Eschborn, Germany) was employed under isocratic conditions with 10% acetonitrile in water (*v*/*v*) as mobile phase A, and pure acetonitrile as mobile phase B. The eluent flow rate was 0.2 mL min^−1^, and the injection volume was 5 μL in all cases. For malathion, the mobile-phase composition was 40% A and 60% B, while for chlorpyrifos, it was 20% A and 80% B. Both OPs were detected at 200 nm. Under these experimental conditions, the retention times were 3.07 ± 0.05 min and 2.53 ± 0.05 min for malathion and chlorpyrifos, respectively. The limit of detection of the method used was 1 × 10^−7^ mol dm^−3^.

### 2.4. Principal Component Analysis

Principal component analysis (PCA) and principal component (PC) linear regression were performed using Scikit-learn built-in functions. Different levels/ranges of the considered input variables were scaled using the StandardScaler function in order to prepare the data for the statistical analysis.

### 2.5. Real-Samples Analysis

For real samples, lemon juice and mint extract were used. Because malathion is mainly used for lemon crop treatment, and chlorpyrifos is used for mint field preservation, we chose these extracts as reals samples. Juice pressed from 1 lemon (75 g) was diluted with 500 mL of tap water (pH = 4.5) and spiked with malathion to achieve the desired concentrations, which was followed by filtration. For mint extract preparation, 7 g of mint leaves (*Mentha spicata*) was mixed with 45 mL of 50% ethanol and left for 72 h at room temperature. After that, the extract was filtered and diluted with 200 mL of 50% ethanol (pH = 6.0). Finally, the mint extract was spiked with the desired amount of chlorpyrifos and consecutively filtered through a nylon filter. Prepared solutions were used in real-samples investigation.

### 2.6. Adsorbent Regeneration

The adsorbent regeneration filter was achieved by washing the modified filter with 5 mL of absolute ethanol for 1 min.

## 3. Results and Discussion

### 3.1. Material Properties

**Figure 1:**
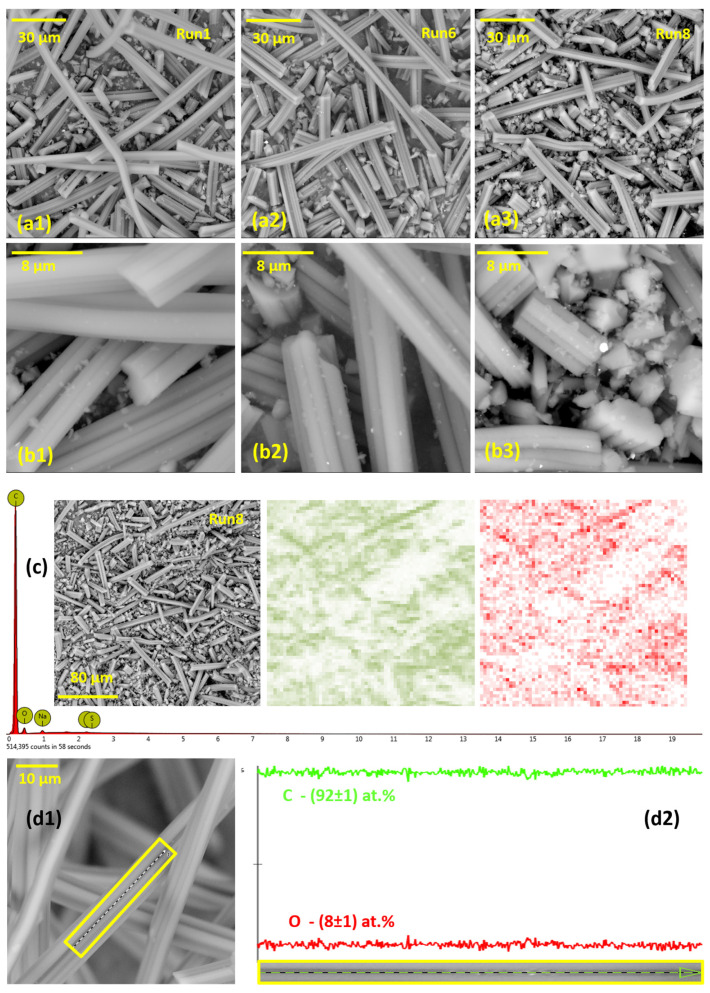
SEM images of samples Run1, Run6, and Run8 at two different magnifications and the EDX map of the Run8 sample with two major elements (C and O) (from (**a1**–**b3**)). EDX spectra (**c**) show only the additional presence of Na and S in the samples. Small debris observed on the fibers are due to the milling procedure, as explained in [43]. (**d1**,**d2**) show the selected region for the line EDX scan along a single fiber of the Run1 sample and the corresponding C and O distribution (Na and S are below 1 at.%) (resolution 512 pixels; three consecutive scans were averaged).

To investigate the samples’ morphology, we used SEM. The SEM micrographs of samples Run1, Run6, and Run8 are presented in Figure 1 (from a1 to b3). The presented micrographs show that the morphology of all the investigated samples is the same and reflects the morphology of precursor viscose fibers, in agreement with our previous findings [20,41]. Namely, in the mentioned works, activated carbon fibers were produced by the carbonization of viscose fibers, either impregnated or without the impregnation step. In both cases, just like we find here, the morphology of the precursor was preserved. Thus, while passing through the carbonization and activation steps, as described in Table 1, fibers keep the morphology of the precursor (see Ref. [42] for SEM micrograph of the precursor), while only a shrinkage of the fiber diameter can be observed.

Upon the carbonization and activation, the fiber widths are around 8 μm, while the flower-like cross-sections (see Figure 1(b3)) are the result of the spinning process during precursor production. The length of the fibers after the milling step is 100–150 μm, while there is a lot of smaller debris of a few microns in length. While the presented finding might seem trivial, it is essential for the further analysis of the material performance. Namely, for the entire series of designed adsorbents, the morphology is not a parameter that could cause intra-series differences in the adsorption performance.

Using EDX, the chemical composition of the produced activated carbon fibers was determined (Table 2). C was found as a major element in all the samples, with roughly 7 at.% of oxygen and traces of Na and S, likely originating from the precursor. It is not surprising that the chemical composition does not show a significant intra-series variation, as the carbonization temperature was the same for all the samples, while the maximum activation temperature was also very close to the carbonization temperature. In all the samples, uniform elemental distribution was observed (Figure 1c), which is not surprising, as the precursor has a uniform chemical composition, while there was no impregnation agent with phase separation that could occur during the carbonization/activation. The overall elemental distribution is not only uniform at low magnification (Figure 1c), but also the elemental distribution along a single fiber (Figure 1(d1,d2)), with the variation under 1 at.% along approx. 35 μm of the fiber length. The main elements (carbon and oxygen) are expected to influence the adsorption process primarily through the specific interactions with the studied pesticides. Considering that chlorpyrifos has an aromatic moiety, it can not only interact with the sp^2^ domains (graphitic) on the carbon surface, but also with oxygen functional groups via dipole interactions. Malathion is a polar aliphatic molecule, and so the interaction with hydrophobic sp^2^ domains in carbon structures is not likely.

**Figure 2:**
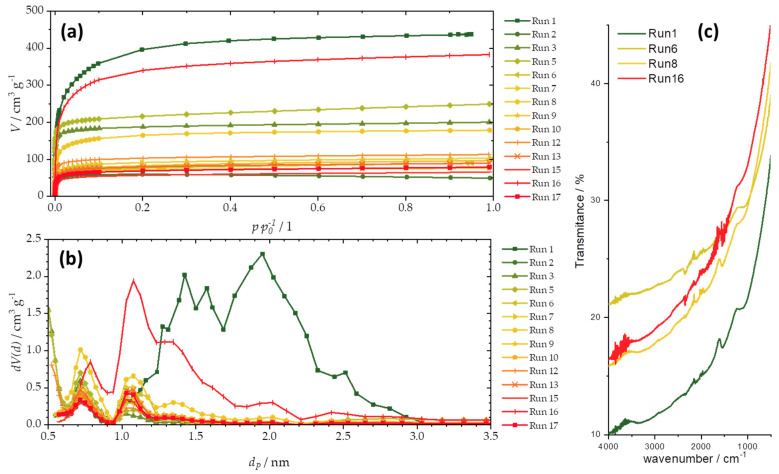
(**a**) Adsorption isotherms, (**b**) pore size distributions, and (**c**) ATR-FTIR spectra of selected carbon materials (FTIR spectra have a high degree of similarity, so the spectra are not given for the entire series).

Figure 2a presents N_2_ adsorption isotherms, while Figure 2b gives the derived pore size distributions (PSDs) for the entire series of samples. The textural properties of the samples are summarized in Table 3, including the total pore volume (*V*_tot_), average pore diameter (*d*_mean_), and specific surface using the BET method (*S*_BET_). The produced activated carbon fibers are dominantly microporous, with only one sample containing pores entering the mesopore domain (i.e., pores with diameters above 2 nm) (Run1, Figure 2b). The considered properties vary by one order of magnitude, suggesting the successful tuning of textural properties by the DoE protocol. Based on the obtained results, the activation temperature, followed by the activation time, play the key role in obtaining samples with higher *S*_BET_.

Considering that the carbonization temperature was the same for all the samples, surface functional groups were also similar for all the samples. Functional groups were probed using ATR-FTIR, and largely featureless spectra were obtained with only a few characteristic bands (Figure 2c). This finding is also in line with the relatively small variations in the oxygen elemental content found using EDX. From the presented FTIR spectra, it can be seen that the bands do not differ significantly in their positions or intensities. Only two bands stand out: those in the spectral ranges of 1606–1633 cm^−1^ and 1242–1287 cm^−1^. The first mentioned band could be associated with in-plane vibrations of the sp^2^ hybridized C=C bonds, while the second originates from C–OH vibrations [44,45]. We note that, as previously discussed [42], the structural disorder in all the samples was the same (as derived from Raman spectroscopy data), as it was dependent only on the temperature at which the samples were carbonized, which is common for all the samples in the series.

### 3.2. Adsorption of OPs

#### 3.2.1. Adsorption Kinetics

The malathion and chlorpyrifos adsorption kinetics was investigated in batch adsorption experiments, and the data were processed by fitting experimental data points into equations corresponding to two frequently used kinetic models—pseudo-first-order (Equation (1)) and pseudo-second-order (Equation (2)) kinetics [46]:

(1) $$q_{t}=q_{e}\left(1-e^{-k_{1}t}\right)$$

(2) $$q_{t}=\frac{k_{2}q_{e}^{2}t}{1+k_{2}q_{e}t}$$

In the equations above, *q_t_* and *q_e_* are the adsorbed amounts of OPs in a given moment of time and the equilibrium adsorption capacity, respectively. The rate constants are *k*_1_ and *k*_2_ for the pseudo-first-order and pseudo-second-order kinetics, respectively.

**Figure 3:**
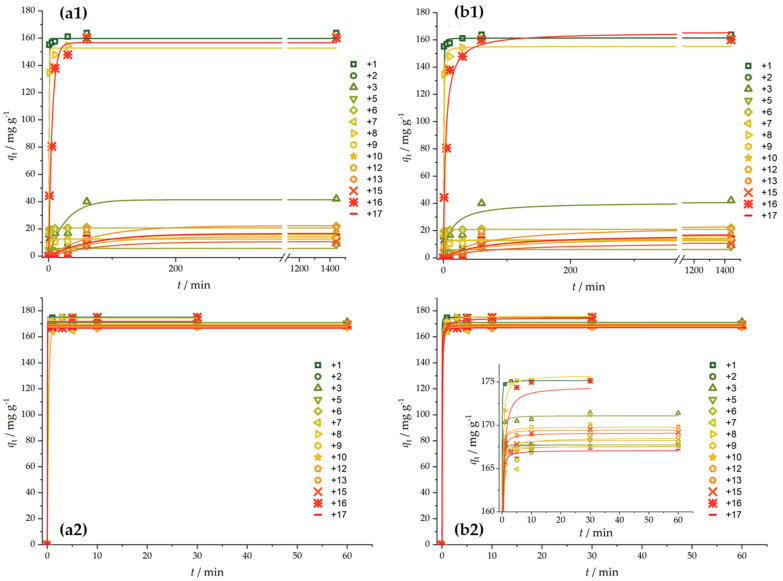
Kinetics of malathion (**a1**,**b1**) and chlorpyrifos (**a2**,**b2**): (**a**) pseudo-first reaction order, (**b**) pseudo-second reaction order. Pesticide concentrations were 5 × 10^−4^ mol dm^−3^, and adsorbent loading was 1 mg mL^−1^. Experimental uncertainties for presented data points are within 5%.

The experimental data and corresponding fits are presented in Figure 3. The obtained equilibrium adsorption capacities and rate constants are summarized in Supplementary Information, Tables S1 and S2. There is a striking difference between malathion and chlorpyrifos adsorption in terms of the adsorption rate, as chlorpyrifos adsorption is much faster than malathion adsorption. Moreover, for malathion, several activated carbon fibers in the studied series performed quite well (Run1, Run8, Run16, and partially Run3), while Run 6 showed a low equilibrium adsorption capacity but high malathion adsorption kinetics. Considering the textural properties shown in Figure 2, it seems that a high adsorption capacity and fast malathion adsorption kinetics require pores larger than 1.2 nm, as this is the range in which the best-performing samples (Run1, Run8, Run16) significantly differ from all the others. In contrast, all the studied adsorbents collect chlorpyrifos exceptionally fast, and equilibrium is reached practically within 10 min of contact. Such fast adsorption made the measurements of *q*_t_ practically impossible for *t* < 1 min, making the use of other kinetic models, such as the inter-particle diffusion model, inappropriate. Namely, there is a lack of experimental points that could cover different time domains corresponding to different processes dominating adsorption.

**Figure 4:**
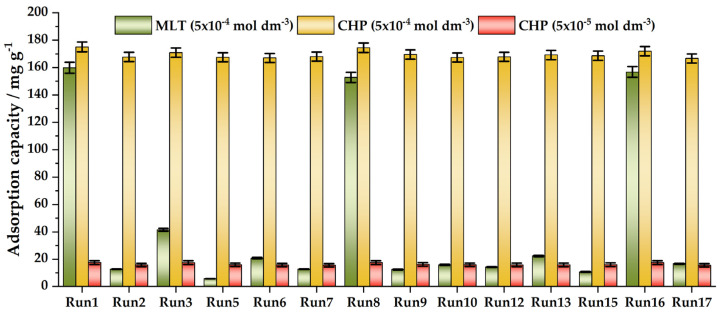
Adsorption capacities for malathion and chlorpyrifos for adsorbent loading of 1 mg mL^−1^.

Figure 4 assembles the obtained equilibrium adsorption capacities for a malathion concentration of 5 × 10^−4^ mol dm^−3^ and chlorpyrifos concentrations of 5 × 10^−5^ mol dm^−3^ and 5 × 10^−4^ mol dm^−3^. As can be seen, the studied adsorbents perform rather well for chlorpyrifos, irrespective of the concentrations. However, for malathion, only three samples have adsorption capacities similar to chlorpyrifos. These are the samples Run1, Run8, and Run16—those with higher *V*_tot_, *d*_mean_, and *S*_BET_. Pore diameter seems quite important. For example, Run5 has a higher *V*_tot_ and *S*_BET_ than Run8 (Table 3), but a much lower *d*_mean_. Run7–9, Run13, and Run16 have the same *d*_mean_, but Run8 and Run16 have the largest *V*_tot_ among these samples and, thus, performed the best.

#### 3.2.2. Adsorption Isotherms

**Figure 5:**
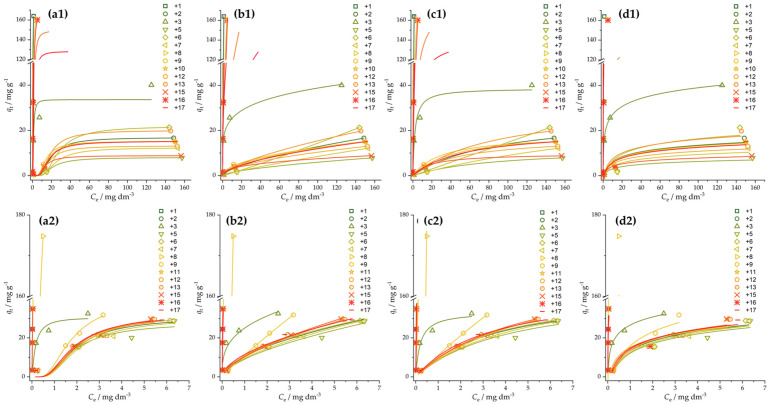
Adsorption isotherms for malathion (**a1**,**b1**,**c1**,**d1**) and chlorpyrifos (**a2**,**b2**,**c2**,**d2**): (**a**) Dubinin–Radushkevich, (**b**) Freundlich, (**c**) Langmuir, and (**d**) Temkin isotherms. Adsorbent loading was 1 mg mL^−1^. Experimental uncertainties for presented data points are within 5%.

The thermodynamics of the malathion and chlorpyrifos adsorption process was investigated by constructing adsorption isotherms and fitting experimental data into several adsorption isotherm models, which reveal different aspects of the adsorption process. We used the Freundlich (Equation (3)), Langmuir (Equation (4)), Temkin (Equation (5)), and Dubinin–Radushkevich (Equation (6)) models. The experimental data points and the corresponding fits are presented in Figure 5, while the fitted parameters are summarized in Supplementary Information, Tables S3–S6.

(3) $$q_{e}=K_{F}{C_{e}}^{1/n}$$

(4) $$q_{e}=\frac{q_{max}K_{L}C_{e}}{1+K_{L}C_{e}}$$

(5) $$q_{e}=\frac{RT}{b_{T}}lnK_{T}C_{e}$$

(6) $$q_{e}=q_{DR}e^{-K_{DR}{Ɛ}^{2}}$$

The used parameters are as follows: *q*_e_ (mg g^−1^)—equilibrium adsorption capacity; *C*_e_ (mg dm^−3^)—equilibrium adsorbate concentration; *K*_F_ (mg g^−1^ (mg dm^−3^)^1/n^) and *n*—Freundlich constants; *K*_L_ (dm^3^ mg^−1^) and *q*_max_ (mg g^−1^)—Langmuir constant and theoretical maximum adsorption capacity of the monolayer, respectively; *b*_T_ (J g mol^−1^ mg^−1^) and *K*_T_ (dm^3^ mg^−1^)—Temkin isotherm constants; *q*_DR_—maximum adsorption capacity; *K*_DR_ (mol^2^ J^−2^)—constant associated with the mean free adsorption energy per mole of adsorbent, *ε* = RT × ln(1 + 1/*C*_e_).

Several adsorbents show a high affinity towards both malathion and chlorpyrifos, making the construction of complete adsorption isotherms difficult due to limitations in chlorpyrifos solubility, while the fitting in these cases is not highly reliable. Similar to the difference in the adsorption kinetics, the thermodynamics of malathion and chlorpyrifos adsorption is quite different. The *n* value of the Freundlich isotherm model is greater than 1 in all cases, indicating that adsorption is a favorable process. Constants indicating an affinity towards adsorption, those from the Temkin, Langmuir, and Freundlich adsorption isotherms, suggest that chlorpyrifos adsorption is more favored than malathion adsorption. The Freundlich isotherm model describes most of the experimental results. Thus, the adsorption is likely physisorption, with a heterogeneous surface of carbon materials. Moreover, based on the Dubinin–Radushkevich model (i.e., the calculated adsorption free energy per mole of adsorbent (*E* = (2K_DR_)^−1/2^ < 8 kJ mol^−1^)) (Table S6, Supplementary Information), it can be seen that the adsorption process is physisorption, which means that there was no chemical bond formation between the OPs and investigated activated carbon fibers.

### 3.3. PCA Analysis

PCA and PC regression were further used to allow feature selection and to study the influence of the synthesis conditions and material properties on the adsorption capacity for both malathion and chlorpyrifos. We note that multiple regression was considered in the analysis of the data during our work, but because a high correlation of various input features was found in the PCA, we found regression based on PC components better suited for this analysis. Therefore, the reported discussion of the additive effects is based on PC regression analysis. The choice of the levels of variables related to the material synthesis conditions is explained in Section 2.1, while all the other variables (material properties and adsorption performance) were determined experimentally.

**Figure 6:**
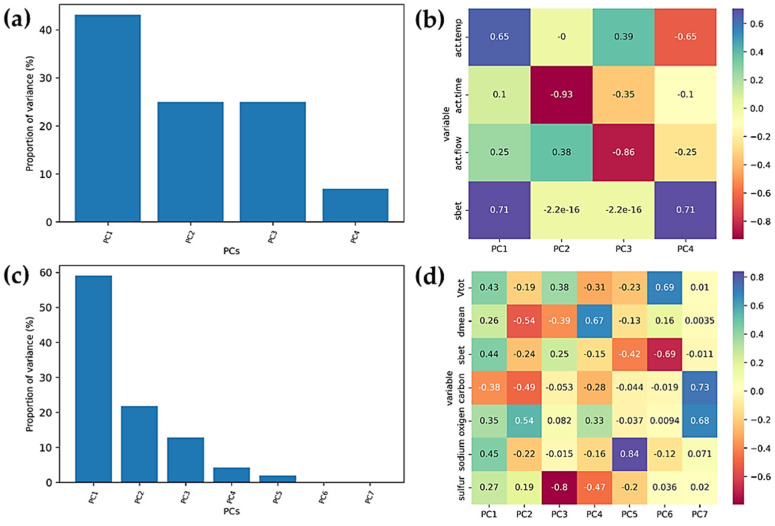
(**a**,**c**) PC variance proportion and (**b**,**d**) heatmap plot of input feature contributions in the PCs for two considered cases. PCA results for case 1 (**a**,**b**)—synthesis conditions are the main input features, and case 2 (**c**,**d**)—material properties are the input features.

As a starting point, 10 input features were considered—those assembled in Table 1, Table 2 and Table 3. After scaling the data and performing PCA, the first three principal components were shown to account for over 80% of the variance (Supplementary Materials, Figure S1). However, a high correlation of multiple features was found in PC1. Therefore, further consideration was carried out by dividing the input features into two sets: the first contained the synthesis conditions (activation temperature, gas flow rate, and activation time) and material surface (*S*_BET_), while the other set included all the material properties. Figure 6 shows the variance contribution of PCs and the heatmap plot of the variable input contribution to each PC for both cases considered.

Based on the PCA, we can see that, in the first case, in which the synthesis conditions are considered, the activation temperature and sample surface size are primary and correlated features in PC1, and the activation time is the main contributor to PC2. In case 2, in which seven material properties are considered as the input features, there is still a correlation of multiple input features in PC1, while PC2 is significantly correlated to the oxygen concentration and negatively correlated to the carbon concentration and pore diameter.

Linear regression was used to consider the performance of the PCs in both cases for the prediction of the malathion and chlorpyrifos adsorption capacities (Figure 4; pesticide concentrations: 5 × 10^−4^ mol dm^−3^). We see that, in both cases, we obtained a better performance for chlorpyrifos. Taking material properties as a starting point for PCA (case 2) outperforms other studied approaches and provides significantly lower mean square errors (MSEs) (Table 4). Thus, linking the synthesis conditions and/or material properties to the material performance for OP removal seems to be a plausible way to rationalize synthetic routes for obtaining high-performance adsorbents. We note that the presented results should be considered as a starting point for building up powerful predictive models, which we plan to establish with the growth of our materials library.

### 3.4. Selective Removal of Pesticides—Adsorption from Mixtures

**Figure 7:**
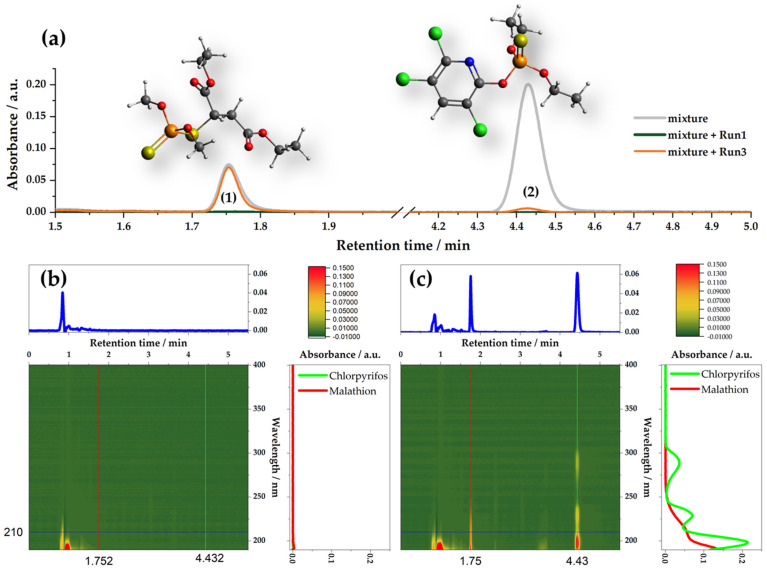
(**a**) UPLC chromatogram of the malathion + chlorpyrifos mixture (5 × 10^−5^ mol dm^−3^) before and after the adsorptive removal using Run1 and Run3 samples: adsorbent loading was 1 mg mL^−1^, and wavelength was 200 nm; peak 1 corresponds to malathion, peak 2 corresponds to chlorpyrifos; (**b**) PDA signal of the malathion + chlorpyrifos mixture after the adsorptive removal of pesticides using the Run1 sample, with extracted chromatogram at 210 nm (at which malathion and chlorpyrifos have the same absorption coefficients), and spectra for retention times 1.75 and 4.43 min; (**c**) the same as for (**b**) but with the sample Run3 as adsorbent.

In the presented series of adsorbents, there are distinct differences in the adsorption performance, where some materials adsorb both malathion and chlorpyrifos, while all materials perform very well for chlorpyrifos removal. Considering that the used OPs are structurally different, the question is whether the adsorption of one OP affects the adsorption of the other one. To investigate the performance of activated carbon fibers towards removing malathion and chlorpyrifos in mixtures, we studied the removal of OPs from the mixture containing 5 × 10^−5^ mol dm^−3^ of malathion and chlorpyrifos, using the adsorbent dose of 1 mg mL^−1^. The results are summarized in Table S7, Supplementary Information. Figure 7 presents the comparison of two selected samples, Run1 and Run3, towards the removal of malathion and chlorpyrifos from the mixtures. Sample Run1 performs well for both OPs (Figure 4 and Table S2, Supporting Information; adsorption capacity for chlorpyrifos 175 mg g^−1^, and for malathion 165 mg g^−1^, while sample Run3 performs well for chlorpyrifos and much lower for malathion (Figure 4 and Table S2, Supporting Information; adsorption capacity for chlorpyrifos 171 mg g^−1^, and for malathion 41 mg g^−1^). As can be seen, Run1 practically completely removes both pesticides (100% uptake, Table S7). Run3 removes chlorpyrifos to a high degree (although less than Run1, uptake is 96.9%), but only 20% of malathion (Table S7). We believe that this is a combination of two factors—the different specific surface areas (Table 3) and different adsorption kinetics of these two pesticides (Figure 3). The sample Run1 has a much larger specific surface and five times larger average pore diameter than Run3, allowing the adsorption of both pesticides due to the availability of a number of adsorption sites, while Run3 has a lower specific surface and much narrower pores. As chlorpyrifos adsorption is faster, it quickly occupies the available surface so that malathion cannot adsorb. Considering, for example, the fitting of the adsorption isotherms using the Freundlich model, the affinity of adsorbents is significantly higher towards chlorpyrifos (Table S3, Supplementary Information) compared to malathion. This leads to the conclusion that once chlorpyrifos is adsorbed onto the surface, it cannot be displaced by malathion. We note that the adsorption performance towards individual pesticides (Figure 4) reflects well on their adsorption performance in the mixture of pesticides (Table S7, Supplementary Information). Thus, it seems that by tailoring the synthetic conditions, which ultimately reflect the material properties, it is possible to obtain adsorbents that selectively remove certain compounds. Naturally, in the case of OPs, the target is to remove as many contaminants as possible, irrespective of their chemical structures. However, considering a broader perspective, particularly in the case of food processing and food safety, it is vital that contaminants are removed while essential nutrients remain in the treated samples, and that adsorbents can operate in complex matrices. This issue is highly relevant, as many contaminants, such as OPs, can be transferred from the original sources into food products. For liquid samples, adsorption seems to be an easy additional (filtration) step to be added in the processing stage, which could significantly improve the quality and safety of the final products if the selective removal of contaminants is possible.

The advantages of using adsorption are clear over aggressive chemical or physical treatments to remove contaminants, which could negatively impact the final product’s nutritional value. Food processing nowadays includes various chemical and physical methods of food treatment using bleach, chlorine dioxide [47], ultraviolet (UV) radiation [48], and peracetic acid [49]. Even though their effects on cleansing food are proven, side effects of their use are evident. Stout et al. showed that the use of bleaching agents leads to the degradation of vitamins and carotenoids in whey protein [50], meaning that the application of bleach in food processing changes the chemical composition of food itself. Chlorine dioxide has similar disinfectant properties to bleach, but it is very unsafe for use in large amounts because it can damage red blood cells and the lining of the gastrointestinal tract. UV radiation has a high oxidation power, and it could cause changes in food taste and discolor it. Furthermore, Li et al. demonstrated that by exposing malathion to UV radiation, it degrades to its more toxic form—malaoxon—making the problem larger [51]. Peracetic acid is corrosive to the eyes, skin, and respiratory tract, making it unsafe to use. Moreover, using it in food processing could affect the content of antioxidant compounds in the food, as its disinfectant activity is based on the release of active oxygen [52].

As the treatment of food with carbon materials does not affect the food itself, only the OP on its surface, and does not cause the formation of more toxic products, it is safe to consider its use during food processing instead of the ones listed above. Moreover, activated carbon has a high potential for application in food packaging, aiming to maintain food quality and control food safety [53].

In the next step, we show that the presented activated carbon fibers can effectively remove the studied OPs from realistic food samples.

### 3.5. Application of Investigated Materials in Food Processing—Filtration Step for Pesticide Removal

In this section, we demonstrate that the presented activated carbon fibers can be used for the removal of chlorpyrifos and malathion from real samples using filtration. In particular, we focused on removing malathion from lemon juice, as lemons are frequently treated by malathion. In addition, we analyzed the removal of chlorpyrifos from mint ethanol extract, in which chlorpyrifos could be found due to the relatively high solubility in ethanol compared to water.

#### 3.5.1. Applications in Real Samples

To investigate the performance of the best-performing materials in the series for the real-sample treatment, lemon juice and mint extract spiked with 5 × 10^−5^ mol dm^−3^ malathion and chlorpyrifos, respectively, were treated with the materials under the dynamic conditions, using modified nylon filters, as described in Section 2.3. For comparison, the adsorption of malathion and chlorpyrifos from deionized water and 50% ethanol, respectively, were investigated under identical conditions. The results are summarized in Table 5.

From Table 5, it can be seen that the investigated materials show comparable uptakes in real samples, as in water and ethanol. The influence of the matrix of the real sample in the analyzed samples was not pronounced and did not interfere with the performance of the investigated adsorbents.

#### 3.5.2. Material Regeneration and Reuse

**Figure 8:**
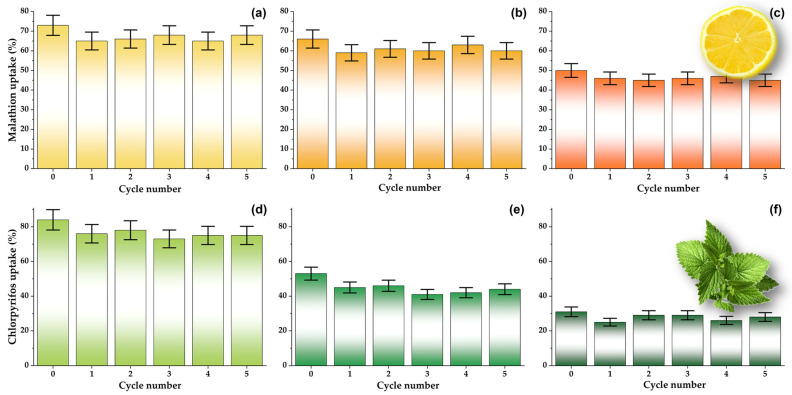
Regeneration of 0.5 mg mL^−1^ of Run1 (**a**,**d**), Run8 (**b**,**e**), and Run16 (**c**,**f**) after the adsorption of 5 × 10^−5^ mol dm^−3^ malathion in lemon juice (**a**,**b**,**c**) and 5 × 10^−5^ mol dm^−3^ chlorpyrifos in mint ethanol extract (**d**,**e**,**f**).

To investigate the possibility of material regeneration, samples Run1, Run8, and Run16 were used for the adsorption under the dynamic conditions for malathion and chlorpyrifos removal from real samples, as described above. After the first round of adsorption experiments, filters modified with material were washed with 5 mL of absolute ethanol for 1 min. Subsequently, filters were used again under the same experimental conditions. This cycle was repeated five times, and the results are presented in Figure 8.

From the data presented in Figure 8, it can be concluded that the investigated material can be successfully regenerated for at least five cycles under the given experimental conditions. Namely, after a small drop in performance between the initial adsorption and the first cycle, the malathion and chlorpyrifos uptakes remained nearly constant in the subsequent five regeneration–adsorption cycles. The presented data strongly emphasize the potential economic aspect of carbon-based filter application in food processing.

## 4. Conclusions

In the present work, a series of viscose-derived activated carbon fibers was produced using the Design of Experiments protocol to set the parametric space for the activation step, allowing for the systematic tuning of the material properties. As a result, highly effective adsorbents for malathion and chlorpyrifos removal were obtained. Both malathion and chlorpyrifos were found to be physisorbed on the studied activated carbon fibers. All the studied materials performed very well for chlorpyrifos removal, giving adsorption capacities above 150 mg g^−1^. The materials with higher surface areas, pore volumes, and larger pore diameters all activated at higher temperatures (870 °C) and also performed as excellent adsorbents for malathion. Moreover, a higher affinity of adsorbents towards chlorpyrifos also resulted in fast adsorption kinetics, where equilibrium was achieved within 10 min of contact with OP-containing solutions. By carefully tuning the carbon materials’ properties, it was possible to selectively remove chlorpyrifos in the presence of malathion. These results have very important implications for further developments. Namely, the problems of complex matrices in food processing can be alleviated by proper material design, allowing the selective adsorptive removal of contaminants while not affecting the nutritive value of food like traditional aggressive chemical and physical treatments. As an example, here, we have demonstrated that the produced carbons can be used for malathion removal from lemon juice and chlorpyrifos removal from mint ethanol extracts, followed by several regeneration cycles, without apparent adsorption performance loss. Thus, precisely tailored carbon materials could be used to process liquid samples used in the food industry to remove contaminants and improve food safety and quality effectively. Considering future perspectives, the present work shows that by using different data-based models, such as PCA and PC regression, it is possible to link the material synthesis conditions and/or material properties to the adsorption performance. Thus, in principle, it is possible to devise models of different complexity that could provide optimal synthetic routes for carbon material for the adsorptive removal of targeted pollutant(s). It is easy to perceive the importance and the possibilities of such an approach; for example, using geographic and environmental data to tackle pollutants that are problematic for a given geographic region. To build a model that can be fed to machine learning models or artificial intelligence, it is essential to generate a sufficient amount of highly reliable data for the training, which requires a systematic approach to material synthesis, careful and in-depth characterization, and standardized protocols for material performance assessment.

## Figures and Tables

**Table 1:** Experimental parameters for the activation of studied series of carbon materials. Carbonization was conducted at 850 °C with a heating rate of 1 °C min^−1^. Missing sample numbers correspond to repetitions of center point Run10.

Sample	Activation Temperature	Activation Time	CO_2_ Flow Rate
Run1	870 °C	180 min	80 L h^−1^
Run2	670 °C	180 min	10 L h^−1^
Run3	870 °C	30 min	80 L h^−1^
Run5	870 °C	30 min	10 L h^−1^
Run6	670 °C	30 min	80 L h^−1^
Run7	670 °C	30 min	10 L h^−1^
Run8	870 °C	180 min	10 L h^−1^
Run9	670 °C	180 min	80 L h^−1^
Run10	770 °C	105 min	45 L h^−1^
Run12	770 °C	180 min	45 L h^−1^
Run13	770 °C	105 min	80 L h^−1^
Run15	670 °C	105 min	45 L h^−1^
Run16	870 °C	105 min	45 L h^−1^
Run17	770 °C	105 min	10 L h^−1^

**Table 2:** Elemental composition of studied carbon materials obtained using EDX (average of four individual spot measurements).

Sample	Carbon (at.%)	Oxygen (at.%)	Sodium (at.%)	Sulfur (at.%)
Run1	91.88	7.60	0.45	0.07
Run2	92.55	7.18	0.21	0.06
Run3	92.13	7.58	0.27	0.02
Run5	92.39	7.28	0.28	0.05
Run6	93.39	6.31	0.26	0.05
Run7	93.63	6.11	0.23	0.03
Run8	92.24	7.38	0.33	0.05
Run9	92.29	7.38	0.27	0.06
Run10	93.08	6.65	0.26	0.01
Run12	91.83	7.91	0.22	0.05
Run13	92.60	7.14	0.23	0.03
Run15	93.99	5.79	0.20	0.02
Run16	92.35	7.33	0.30	0.02
Run17	93.13	6.65	0.21	0.02

**Table 3:** Textural properties of studied carbons: *V*_tot_—total pore volume; *d*_mean_—average pore diameter; *S*_BET_—specific surface using BET method.

Sample	*V*_tot_/cm^3^ g^−1^	*d*_mean_/nm	*S*_BET_/m^2^ g^−1^
Run1	0.547	1.951	1791
Run2	0.074	1.029	227
Run3	0.247	0.479	659
Run5	0.307	0.479	746
Run6	0.108	0.718	259
Run7	0.122	0.718	309
Run8	0.212	0.718	372
Run9	0.090	0.718	250
Run10	0.115	1.077	277
Run12	0.135	0.524	353
Run13	0.106	0.718	270
Run15	0.078	1.029	192
Run16	0.452	0.718	1141
Run17	0.097	0.574	232

**Table 4:** Mean square errors for linear regression model trained with various numbers of PCs from two cases described in the text: case 1, in which synthesis conditions and *S*_BET_ are taken as input features for PCA, and case 2, in which 7 material properties are considered as input features for PC (*q*_MLT_—malathion adsorption capacity, *q*_CHP_—chlorpyrifos adsorption capacity).

	Case 1		Case 2	
	MSE(*q*_MLT_) /mg g^−1^	MSE(*q*_CHP_) /mg g^−1^	MSE(*q*_MLT_) /mg g^−1^	MSE(*q*_CHP_) /mg g^−1^
PC1	63.35	2.33	43.77	0.94
PC1-2	56.5	2.26	27.26	0.59
PC1-3	56.19	3.06	187	6.65

**Table 5:** Typical uptakes for 5 × 10^−5^ mol dm^−3^ malathion in deionized water and lemon juice and 5 × 10^−5^ mol dm^−3^ chlorpyrifos in 50% ethanol and mint ethanol extract. Presented results were obtained when 0.5 mg mL^−1^ of adsorbents were used under dynamic conditions at 20 °C.

Material	Uptake (%)			
	Malathion in Deionized Water	Malathion in Lemon Juice	Chlorpyrifos in 50% Ethanol	Chlorpyrifos in Mint Ethanol Extract
Run1	81 ± 4	73 ± 5	90 ± 3	84 ± 5
Run8	64 ± 4	66 ± 4	51 ± 4	53 ± 3
Run16	47 ± 3	39 ± 4	34 ± 3	31 ± 3

## Data Availability

The data used to support the findings of this study can be made available by the corresponding author upon request.

## Supplementary Materials

The following supporting information can be downloaded at https://www.mdpi.com/article/10.3390/foods12122362/s1, Table S1: Kinetics parameters for malathion (5 × 10^−4^ mol dm^−3^) and chlorpyrifos (5 × 10^−4^ mol dm^−3^) for pseudo-first-order kinetics, adsorbent dose 1 mg mL^−1^; Table S2: Kinetics parameters for malathion (5 × 10^−4^ mol dm^−3^) and chlorpyrifos (5 × 10^−4^ mol dm^−3^) for pseudo-second-order kinetics, adsorbent dose 1 mg mL^−1^; Table S3: Parameters for malathion and chlorpyrifos adsorption using Freundlich adsorption isotherm, adsorbent dose 1 mg mL^−1^; Table S4: Parameters for malathion and chlorpyrifos adsorption using Langmuir adsorption isotherm, adsorbent dose 1 mg mL^−1^; Table S5: Parameters for malathion and chlorpyrifos adsorption using Temkin adsorption isotherm, adsorbent dose 1 mg mL^−1^; Table S6: Parameters for malathion and chlorpyrifos adsorption using Dubinin–Raduskevich adsorption isotherm, adsorbent dose 1 mg mL^−1^ (MLT—malathion, CHP—chlopryrifos); Figure S1: PC variance proportion (left) and heatmap plot of input feature contributions in the PCs (right) for the case of 10 input variables; Table S7: Adsorptive removal of malathion and chlorpyrifos in the mixture (5 × 10^−5^ mol dm^−3^ of each pesticide; 30 min equilibration time; 25 °C; adsorbent dose 1 mg mL^−1^) given as pesticide uptake.
