# Supplementary material for: Application of Viscose-Based Porous Carbon Fibers in Food Processing—Malathion and Chlorpyrifos Removal
Source: Foods. 2023 Jun 13;12(12):2362. doi: 10.3390/foods12122362 (PMC10297268; doi:10.3390/foods12122362)
Supplement: Supplementary file 1 [file foods-12-02362-s001.zip › foods-2415535-supplementary.pdf]

foods

Article

Application of Viscose-Based Porous Carbon Fibers in Food Processing—Malathion and Chlorpyrifos Removal

Tamara Tasić [1], Vedran Milanković [1], Katarina Batalović [1], Stefan Breitenbach [2,3], Christoph Unterweger [2], Christian Fürst [2], Igor A. Pašti [4,*] and Tamara Lazarević-Pašti [1]

[1] VINČA Institute of Nuclear Sciences—National Institute of the Republic of Serbia, University of Belgrade, Mike Petrovica Alasa 12–14, 11000 Belgrade, Serbia; tamara.tasic@vin.bg.ac.rs (T.T.); vedran.milankovic@vin.bg.ac.rs (V.M.); kciric@vin.bg.ac.rs (K.B.); tamara@vin.bg.ac.rs (T.L.-P.)
[2] Wood K Plus—Kompetenzzentrum Holz GmbH, Altenberger Strasse 69, 4040 Linz, Austria; s.breitenbach@wood-kplus.at (S.B.); c.unterweger@wood-kplus.at (C.U.); c.fuerst@wood-kplus.at (C.F.)
[3] Institute of Chemical Technology of Inorganic Materials (TIM), Johannes Kepler University Linz, Altenberger Strasse 69, 4040 Linz, Austria
[4] Faculty of Physical Chemistry, University of Belgrade, Studentski Trg 12–16, 11158 Belgrade, Serbia
* Correspondence: igor@ffh.bg.ac.rs

Table S1. Kinetics parameters for malathion (5×10^{-4} mol dm^{-3}) and chlorpyrifos (5×10^{-4} mol dm^{-3}) for pseudo-first-order kinetics, adsorbent dose 1 mg ml^{-1}.

Material	Pseudo-first order kinetics							
	q_e (mg g^{-1})		k_2 (min^{-1})		χ^2		R^2	
	malathion	chlorpyrifos	malathion	chlorpyrifos	malathion	chlorpyrifos	malathion	chlorpyrifos
Run1	159.772	175.040	7.28×10^9	7242.4	13.978	0.029	0.996	0.999
Run2	12.657	167.674	2.025	164.902	11.704	0.015	0.635	0.999
Run3	41.425	170.879	0.036	145.493	88.377	0.258	0.601	0.999
Run5	5.756	167.464	1.650	135.333	4.276	0.445	0.476	0.999
Run6	20.777	166.957	2.950	84.953	0.746	1.806	0.988	0.999
Run7	12.688	168.008	1.20×10^5	3.874	4.488	5.013	0.832	0.999
Run8	152.754	174.456	2.128	57473	66.580	2.392	0.980	0.999
Run9	12.448	169.419	1.124	117.977	6.558	0.506	0.760	0.999
Run10	15.859	167.294	0.015	139.604	14.468	0.168	0.732	0.999
Run12	14.258	167.828	0.015	112.966	10.543	0.774	0.750	0.999
Run13	22.393	169.189	0.014	138.148	23.982	0.345	0.764	0.999
Run15	10.691	168.563	0.013	95.282	4.604	0.945	0.790	0.999
Run16	156.692	171.865	0.181	49372	127.362	16.623	0.969	0.997
Run17	16.648	166.658	0.014	111.398	13.972	0.495	0.755	0.999

Table S2. Kinetics parameters for malathion ($5×10^{-4}$ mol dm^{-3}) and chlorpyrifos ($5×10^{-4}$ mol dm^{-3}) for pseudo-second-order kinetics, adsorbent dose 1 mg ml^{-1}.

Materials	Pseudo-Second Order Kinetics							
	q_e (mg g^{-1})		k_1 (min^{-1})		χ^2		R^2	
	malathion	chlorpyrifos	malathion	chlorpyrifos	malathion	chlorpyrifos	malathion	chlorpyrifos
Run1	161.395	175.179	0.134	2.390	6.483	0.003	0.998	0.999
Run2	13.114	167.734	0.246	4.570	10.988	0.007	0.657	0.999
Run3	40.922	171.105	0.002	1.190	74.347	0.138	0.664	0.999
Run5	6.189	167.756	0.238	0.918	3.881	0.245	0.525	0.999
Run6	20.957	167.605	0.586	0.412	0.584	0.826	0.990	0.999
Run7	12.689	168.540	$2.68×10^{44}$	0.215	4.488	3.967	0.832	0.999
Run8	155.292	175.760	0.036	0.252	41.154	0.108	0.988	0.999
Run9	13.453	169.810	0.079	0.688	4.513	0.149	0.835	0.999
Run10	16.782	167.532	$9.94×10^{-4}$	1.130	17.000	0.035	0.685	0.999
Run12	15.111	168.267	0.001	0.610	12.360	0.323	0.707	0.999
Run13	23.766	169.459	$6.34×10^{-4}$	0.994	28.060	0.174	0.723	0.999
Run15	11.380	175.179	0.001	0.492	5.361	0.256	0.756	0.999
Run16	165.618	167.734	0.002	0.122	121.284	7.551	0.970	0.998
Run17	17.655	171.105	$8.79×10^{-4}$	0.629	16.370	0.070	0.713	0.999

Table S3. Parameters for malathion and chlorpyrifos adsorption using Freundlich adsorption isotherm, adsorbent dose 1 mg ml^{-1}.

Materials	Freundlich isotherm							
	K_F ((dm^3 mg^{-1})$^{1/n}$)		*n*		χ^2		R^2	
	malathion	chlorpyrifos	malathion	chlorpyrifos	malathion	chlorpyrifos	malathion	chlorpyrifos
Run1	131.634	427.447	1.222	1.327	1.200	1.404	0.999	0.992
Run2	0.627	10.639	1.526	1.803	0.009	1.772	0.999	0.985
Run3	16.647	25.087	5.441	3.113	3.735	12.394	0.986	0.917
Run5	0.342	9.087	1.613	1.659	0.085	4.773	0.994	0.958
Run6	0.098	11.325	0.923	1.902	0.145	2.517	0.999	0.978
Run7	0.185	9.547	1.204	1.655	0.174	0.549	0.996	0.995
Run8	60.607	284.974	1.487	1.452	6.100	15.588	0.999	0.998
Run9	1.343	11.204	2.068	1.099	1.355	0.058	0.976	0.999
Run10	1.072	13.006	1.893	2.095	0.094	14.413	0.998	0.881
Run12	0.855	10.114	1.846	1.573	0.025	0.814	0.999	0.993
Run13	0.740	10.757	1.516	1.676	$6.49×10^{-4}$	0.157	0.999	0.999
Run15	1.324	10.355	2.659	1.566	0.606	0.193	0.964	0.998
Run16	44.814	520.949	1.282	1.252	11.452	0.597	0.998	0.997
Run17	0.857	11.492	1.750	1.833	0.126	2.239	0.998	0.981

Table S4. Parameters for malathion and chlorpyrifos adsorption using Langmuir adsorption isotherm, adsorbent dose 1 mg ml^{-1}.

Materials	Langmuir isotherm							
	K_L (dm^3 mg^{-1})		q_{max} (mg g^{-1})		χ^2		R^2	
	malathion	chlorpyrifos	malathion	chlorpyrifos	malathion	chlorpyrifos	malathion	chlorpyrifos
Run1	0.057	9.79×10^{-4}	2377.669	1.04×10^{6}	51.800	12.155	0.991	0.930
Run2	0.011	0.281	26.697	45.049	0.177	0.315	0.997	0.997
Run3	0.442	5.038	38.672	33.488	15.938	5.418	0.939	0.964
Run5	0.011	0.229	12.362	45.597	0.231	6.594	0.985	0.942
Run6	1.63×10^{-5}	0.271	9060.294	46.504	0.291	3.446	0.998	0.971
Run7	0.003	0.211	36.070	49.891	0.226	0.659	0.994	0.994
Run8	0.402	0.002	254.409	1.48×10^{5}	1.241	249.801	0.999	0.960
Run9	0.033	0.048	18.150	240.797	0.042	0.067	0.999	0.999
Run10	0.022	0.503	19.621	39.361	0.130	4.028	0.998	0.967
Run12	0.020	0.208	17.308	54.840	0.140	1.384	0.997	0.989
Run13	0.011	0.232	31.698	52.202	0.160	1.449	0.998	0.988
Run15	0.054	0.196	9.797	58.122	0.047	0.632	0.997	0.995
Run16	0.131	9.79×10^{-4}	398.735	1.10×10^{6}	25.103	6.445	0.995	0.963
Run17	0.018	0.297	20.502	46.399	0.034	1.386	0.999	0.988

Table S5. Parameters for malathion and chlorpyrifos adsorption using Temkin adsorption isotherm, adsorbent dose 1 mg ml^{-1}.

Materials	Temkin isotherm							
	K_T (dm^3 mg^{-1})		b_T (J g mol^{-1} mg^{-1})		χ^2		R^2	
	malathion	chlorpyrifos	malathion	chlorpyrifos	malathion	chlorpyrifos	malathion	chlorpyrifos
Run1	100.296	479.958	100.387	224.862	2667.335	14.439	0.520	0.917
Run2	0.738	6.314	793.351	341.343	21.023	6.162	0.711	0.947
Run3	19.194	84.807	481.118	412.619	1.381	1.044	0.995	0.993
Run5	0.815	5.424	1741.088	346.078	5.074	10.864	0.670	0.904
Run6	0.471	12.283	584.517	411.541	62.868	13.147	0.538	0.888
Run7	0.616	5.501	1091.750	336.207	17.146	7.861	0.577	0.932
Run8	78.871	114.082	122.849	62.323	2646.405	750.152	0.505	0.880
Run9	0.754	4.578	817.788	237.338	3.642	18.069	0.935	0.875
Run10	0.914	5.887	885.685	303.901	10.140	4.171	0.820	0.966
Run12	0.868	5.658	1026.504	318.366	8.288	9.401	0.801	0.923
Run13	0.741	8.147	664.411	356.209	29.197	11.131	0.719	0.908
Run15	1.345	6.471	1565.484	328.428	0.500	11.371	0.970	0.908
Run16	39.979	553.185	112.639	235.892	2561.640	20.346	0.516	0.884
Run17	0.720	8.324	848.424	357.100	10.500	9.199	0.817	0.923

Table S6. Parameters for malathion and chlorpyrifos adsorption using Dubinin-Radushkevich adsorption isotherm, adsorbent dose 1 mg ml^{-1} (MLT – malathion, CHP – chlorpyrifos).

Materials	Dubinin-Radushkevich isotherm									
	q_{DR} (mg g^{-1})		K_{DR} (mol^2 J^{-2})		E (J mol^{-1})		χ^2		R^2	
	MLT	CHP	MLT	CHP	MLT	CHP	MLT	CHP	MLT	CHP
Run1	191.450	101.691	7.98×10^{-8}	1.56×10^{-8}	2503.479	5663.389	70.443	0.184	0.987	0.999
Run2	16.849	30.901	4.89×10^{-5}	6.87×10^{-7}	101.124	853.065	0.488	6.428	0.993	0.945
Run3	33.577	30.522	3.01×10^{-7}	2.73×10^{-8}	1288.197	4276.813	46.405	9.068	0.824	0.940
Run5	7.999	28.013	5.89×10^{-5}	6.39×10^{-7}	92.167	884.642	0.383	16.929	0.975	0.850
Run6	21.903	32.334	9.57×10^{-5}	7.71×10^{-7}	72.288	805.232	0.242	5.795	0.998	0.950
Run7	12.365	30.578	7.74×10^{-5}	7.86×10^{-7}	80.382	797.580	0.359	8.619	0.991	0.925
Run8	166.705	216.352	1.40×10^{-7}	2.86×10^{-8}	1886.996	4183.393	45.001	47.771	0.992	0.992
Run9	15.175	41.935	2.61×10^{-5}	6.27×10^{-7}	138.421	892.970	0.223	6.249	0.996	0.957
Run10	15.246	31.160	3.47×10^{-5}	4.10×10^{-7}	120.121	1104.703	0.556	6.276	0.990	0.948
Run12	13.139	31.323	3.92×10^{-5}	6.44×10^{-7}	112.891	880.803	0.482	10.729	0.988	0.912
Run13	19.999	31.590	4.54×10^{-5}	6.42×10^{-7}	104.925	882.395	0.528	9.412	0.995	0.923
Run15	8.811	33.012	2.27×10^{-5}	7.24×10^{-7}	148.264	830.927	0.477	7.540	0.971	0.939
Run16	171.739	113.167	3.59×10^{-7}	1.65×10^{-8}	1180.622	5498.127	132.980	0.893	0.975	0.995
Run17	15.164	32.253	3.91×10^{-5}	6.54×10^{-7}	113.139	874.537	0.332	5.665	0.994	0.953

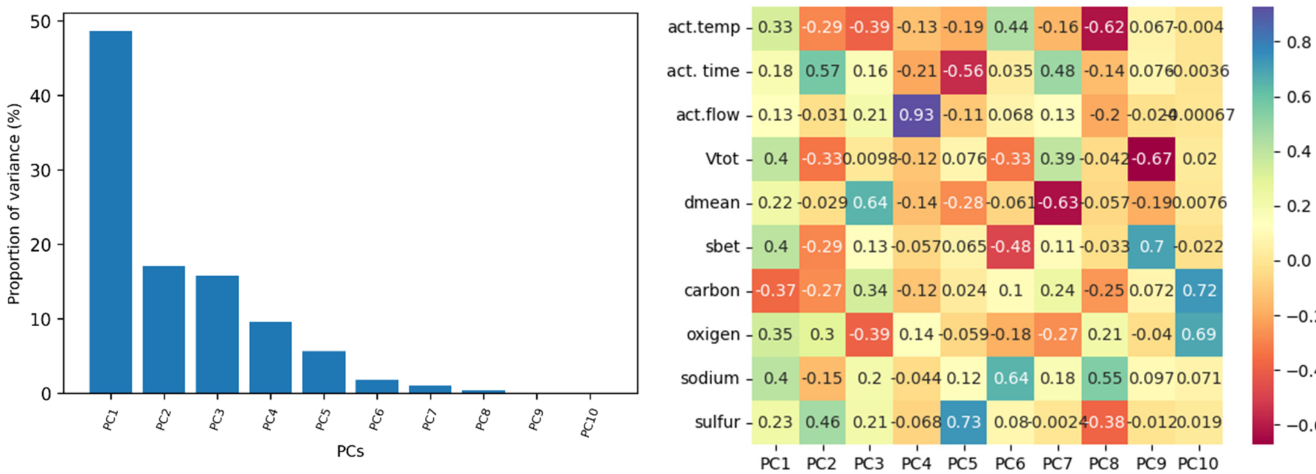

Figure S1. PC variance proportion (left) and heatmap plot of input feature contributions in the PCs (right) for the case of 10 input variables.

Table S7. Adsorptive removal of malathion and chlorpyrifos in the mixture (5×10^{-5} mol dm^{-3} of each pesticide, 30 min equilibration time, 25 °C, adsorbent dose 1 mg ml^{-1}) given as pesticide uptake.

Adsorbent	Malathion uptake (%)	Chlorpyrifos uptake (%)
Run1	100	100
Run2	0	88.00
Run3	19.29	96.90
Run5	0	86.24
Run6	0	87.49
Run7	0	85.80
Run8	100	100
Run9	0	89.22
Run10	0	87.74
Run12	0	87.94
Run13	0	88.73
Run15	0	87.98
Run16	99.10	100
Run17	0	86.64
